# Influenza Virus Isolation for Public Health Surveillance Before, During, and After the COVID-19 Pandemic: Experiences from the New York State National Influenza Reference Center Laboratory

**DOI:** 10.3390/idr18040071

**Published:** 2026-07-10

**Authors:** Amruta Pramod Moghe, Emaly Starrett Leak, Jennifer May Laplante, Kirsten St. George

**Affiliations:** 1 Laboratory of Viral Diseases, Wadsworth Center, New York State Department of Health, Albany, NY 12208, USA; emaly.leak@health.ny.gov (E.S.L.); jennifer.laplante@health.ny.gov (J.M.L.); kirsten.st.george@health.ny.gov (K.S.G.); 2 Department of Biomedical Sciences, University at Albany, State University of New York, Albany, NY 12222, USA

**Keywords:** influenza, annual influenza surveillance, COVID-19 pandemic, vaccine

## Abstract

Background: Influenza viruses can cause mild to severe illnesses. The burden of disease varies widely depending on multiple factors, including the type and subtype of circulating viruses, timing of the season, flu vaccine efficacy and vaccination rates. Influenza viruses are also highly prone to genetic change and rapid spread due to modern human movement patterns, making influenza surveillance vital for public health awareness, guidance, policy, disease mitigation, and annual recommendations on vaccine composition. Methods: A network of three National Influenza Reference Centers (NIRCs) was established in the United States more than 10 years ago to support the Centers for Disease Control and Prevention’s (CDC) Influenza Division with its national influenza surveillance efforts. Located in California, New York, and Wisconsin, they are funded by CDC via a collaborative agreement with the Association of Public Health Laboratories (APHL). The role of the NIRCs is critical to national and global influenza surveillance, providing rapid information on circulating influenza strains from three arms of laboratory testing: (1) the virus isolation project (VIP), (2) next-generation sequencing (NGS), and (3) anti-viral drug resistance testing. Results: Here, we review the data generated in the VIP lab of the New York State (NYS) NIRC before, during, and after the COVID-19 pandemic and discuss its utility in an understanding of disease dynamics and viral evolution, as well as public health policy and decision making during this historic period in health care. Conclusion: Continued preparedness and surveillance are critical to mitigating the impact of evolving influenza viruses.

## 1. Introduction

In temperate climates, including the United States (U.S.), influenza is seasonal with peaks in late fall and winter, although seasonal influenza viruses are detected at low levels outside of respiratory season. The Centers for Disease Control and Prevention (CDC) estimated that influenza caused 9.3–41 million illnesses, 100,000–710,000 hospitalizations, and 4900–51,000 deaths in the U.S. annually between 2010 and 2023 [1,2]. Influenza activity usually begins to increase in October, peaking between December and February, and can last as late as May [3]. The exact timing and duration of the season vary each year and depend on many factors such as the characteristics of circulating strains, weather patterns, how well vaccines cross-protect against circulating viruses, and the proportion of people vaccinated. Given the significance of the disease burden caused by influenza viruses, it is important to monitor the circulating strains each year, both nationally and globally. For this purpose, three National Influenza Reference Centers (NIRCs) were established in the U.S. located in California (California Department of Public Health, Viral and Rickettsial Disease Laboratory), New York (New York State Department of Health, Wadsworth Center), and Wisconsin (Wisconsin State Laboratory of Hygiene), to contribute surveillance data for a comprehensive picture of the antigenic, genetic, and antiviral susceptibility properties of influenza viruses that are circulating and/or emerging in the U.S. Global surveillance was initiated by the World Health Organization’s (WHO’s) Global Influenza Surveillance and Response System (GISRS) in 1952. Since then, the GISRS network has provided detailed data for the biannual selection of strains recommended for inclusion in the Northern and Southern hemisphere influenza vaccines [4].

The Influenza Division at CDC gathers, compiles, and analyzes information on influenza activity year-round in the U.S. It comprehensively monitors the evolving changes in viruses, including minor genetic changes that lead to antigenic differences among viruses (antigenic drift). The Influenza Division also tracks major genetic reassortment events that can lead to novel subtypes’ emergence or new clades of HA and NA genes in humans (antigenic shift), as well as the introduction and spread of new strains into different geographic regions. To mitigate illness, annual vaccination is recommended, and vaccines are updated annually based on surveillance findings and virologic characterization. Antiviral susceptibility monitoring and testing also provide essential information for influenza treatment recommendations, and surveillance, in general, provides an understanding of the impact of influenza across numerous patient groups in the population [5].

The U.S. influenza surveillance system is a joint effort between the CDC and its many partners in state, local, and territorial health departments, public health and clinical laboratories, health care providers, hospitals, clinics, emergency departments, and long-term care facilities. Each respiratory season, public health laboratories from all 50 states and territories participate in influenza testing and provide influenza-positive clinical specimens to their designated NIRC. In the NIRC laboratories, viruses from these positive clinical specimens are isolated and cultured to larger volumes to provide sufficient material for additional biological characterization and testing. They also undergo next-generation sequencing for analysis of the entire viral genome. Assignments vary each season slightly, but the NYS NIRC at the Wadsworth Center is responsible for testing influenza samples from 20 to 23 states in the eastern U.S.

## 2. Methods

Each NIRC follows standardized protocols, developed by CDC, for the isolation and testing of influenza viruses from the submitted positive clinical specimens. From 2017 to 2023, the NYS NIRC VIP lab received positive influenza clinical specimens from 20 to 23 eastern US states.

Submitting state public health laboratories select clinical specimens from patients of varying ages, disease severity, and geographic location, and include a mixture of influenza types and subtypes. All submitting laboratories follow the submission guidance provided each season by CDC and APHL, submitting positive clinical specimens every two weeks to capture recently circulating strains. Guidance to all states was to ship specimens with a Cycle Threshold (Ct) value less than 30 based on Inf A or Inf B testing using the CDC Flu r-RT_PCR Dx panel for season 2018–2018 and 2018–2019. Later, from the 2019–2020 season onwards, the required Ct value was reduced to 28 or lower. Specimen volume requirements also varied each season, but it was recommended to submit no less than 0.30 mL of the original specimen.

During 2017 to 2019, laboratories were directed to submit two specimens of each influenza A/H1pdm09 and A/H3, and four of influenza B biweekly. For 2019 to 2020, this was increased to two influenza A/H1pdm09, three influenza A/H3, and four influenza B. From 2020 to 2022, the influenza B component of these submissions was further specified to include two B/Victoria and two B/Yamagata. From 2022 to 2023, this was further increased to biweekly submissions of four influenza A/H1pdm09, six influenza A/H3, four influenza B/Victoria, and four B/Yamagata. Upon receipt at the NYS NIRC, clinical specimens are accessioned in the Contract Lab Accessioning Web portal (CLAW), and unique CDC identification numbers are generated.

Two cell lines are used to propagate viruses. Influenza A viruses (both A/H1N1pdm09 and A/H3N2) are grown in the MDCK-SIAT cell line (Sigma-Aldrich, Cat No. 0501502, Atlanta, GA, USA) [6] while influenza B viruses are inoculated into the MDCK-ATL cell line (ATCC, IRR item no. FR-926, ATCC IRR, Manassas, VA, USA) [7]. Viruses are isolated in T-75 flasks, with cells seeded at 90–95% confluency. Following inoculation, flasks are checked daily for cytopathic effect (CPE), and cultures are harvested at 48 h post-inoculation for A/H1N1pdm09 and at 72 h post-inoculation for A/H3N2 and B viruses. CPE was recorded by observing the changes in the cell monolayer with a grading system as follows: − = negative, no virus growth, +/− = ~10% infected, 1 = ~25% infected, 2 = ~50% infected, 3 = ~75% infected, and 4 = ~100% infected.

Harvested clinical specimens are tested in hemagglutination assays (HA) to measure the presence of viruses in cultures and HA titers. The HA assay takes advantage of the ability of the influenza virus’s hemagglutinin (HA) protein to agglutinate red blood cells (RBCs). The number of viruses in a sample or harvested cell culture supernatant can be estimated by how much the red cells agglutinate the virus across a range of serial dilutions. Major advantages of the HA assay are its simplicity, speed, and cost-effectiveness. Influenza A/H3N2 virus isolates are titered with (0.75%) Guinea pig RBCs, while A/H1N1pdm09 and influenza B isolates are titered with (0.5%) Turkey RBCs. HA plates are incubated at room temperature for 30 min and 60 min for turkey blood and Guinea pig RBCs, respectively. Harvested viruses in clarified supernatants are then dispensed into several aliquots for distribution to other laboratories within the NIRC for next-generation sequencing, and, together with residual original specimens, to the CDC for further biological testing.

## 3. Results

**Figure 1:**
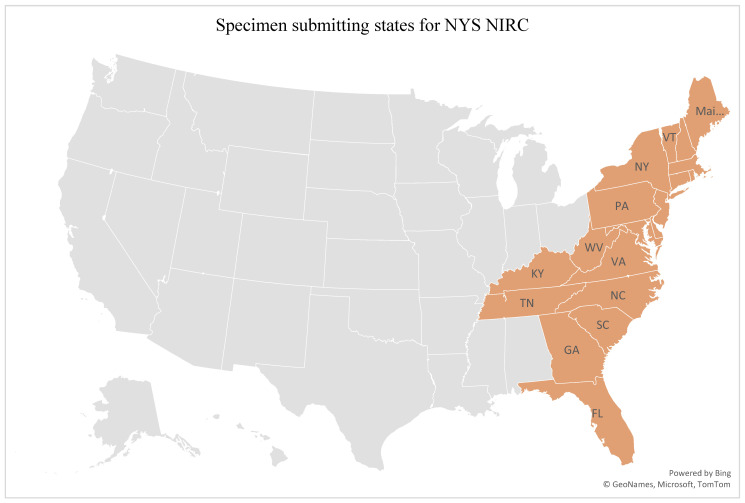
The orange-highlighted states are designated submitters to the NYS NIRC. The two-letter abbreviations in the maps represent different states in the U.S.A. VT = Vermont, ME = Maine, NY = New York, PA = Pennsylvania, WV = West Virginia, VA = Virginia, NC = North Carolina, SC = South Carolina, GA = Georgia, FL = Florida, KY = Kentucky, TN = Tennessee.

To review influenza trends before, during, and after the COVID-19 pandemic, six influenza seasons were selected for study, spanning the seasons from 2017 to 2018 through 2022–2023. During this time, the NYS NIRC received clinical influenza specimens from 20 to 23 states each year, as shown in Figure 1. The CDC/APHL guidance and the actual numbers of submissions each season varied, depending on the circulating viruses that year.

**Figure 2:**
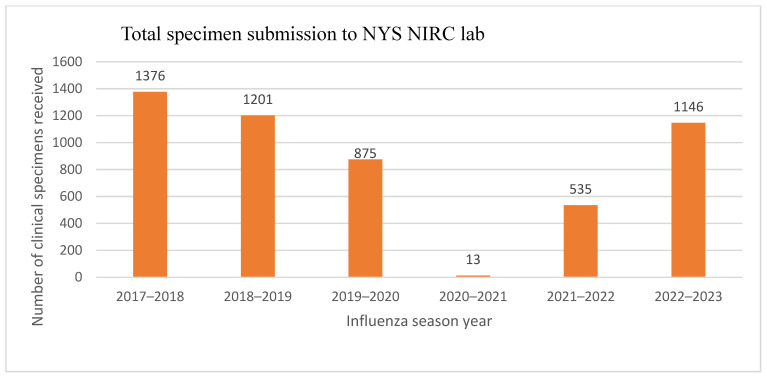
The number of influenza sample submissions to the NYS NIRC over the six-season study period.

Figure 2 shows the total number of specimens submitted to NYS NIRC each season during the study period. During typical influenza seasons before the pandemic (2017–2018 and 2018–2019), more than 1000 clinical specimens were received each season. However, after the onset of the COVID-19 pandemic (late 2019–early 2020), submissions declined, and during the peak of the pandemic (2020–2021), submissions reached their lowest level, corresponding to the historically low levels of reported influenza activity in the U.S. during this time [8]. This reduced activity was likely caused by multiple factors, notably widespread implementation of community mitigation measures such as school closures, social distancing, and mask policies, but also changes in health care-seeking priorities and influenza testing practices [9]. As SARS-CoV-2 activity started decreasing, influenza activity and the concurrent number of influenza submissions to the NIRC increased gradually (2021–2022), approaching usual numbers in the 2022–2023 season.

**Figure 3:**
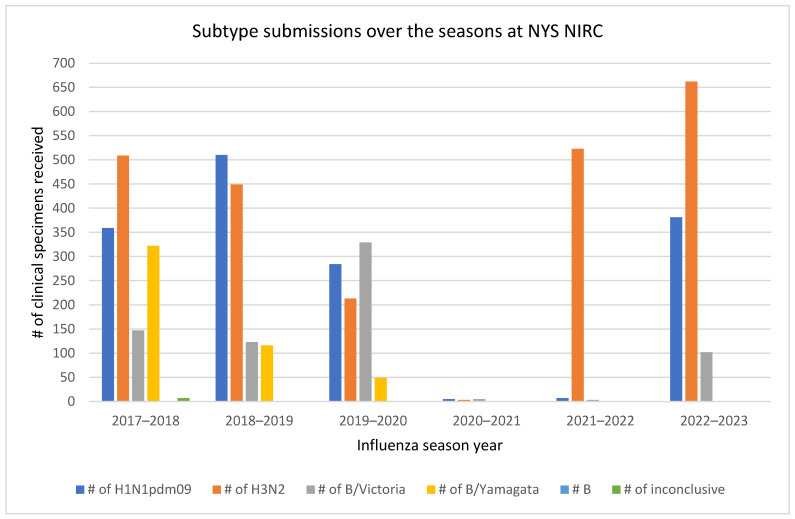
Influenza subtypes submitted to the NYS NIRC during each of the six seasons studied.

Typically, in the U.S. and elsewhere, multiple types and subtypes of influenza may circulate during a season [10]. From 2010 to 2020, influenza A viruses represented 78% of viruses detected, with influenza B comprising 22% [10]. Figure 3 shows the trend of influenza subtype submissions to the NYS NIRC across different seasons. As shown, the influenza A/H3N2 subtype was dominant among submissions for the 2017–2018, 2021–2022, and 2022–2023 seasons, while for season 2018–2019, both A/H3N2 and A/H1N1pdm09 were co-dominant. Influenza B submissions were moderate for the first three seasons of the study period. Influenza B/Yamagata was more prominent than the B/Victoria lineage for season 2017–2018, while B/Victoria submissions were at higher levels during the 2019–2020 season. Notably, from mid-2020 onwards, no B/Yamagata-positive clinical specimens were submitted to the NYS NIRC lab, and based on the available data, that lineage was not detected anywhere in the U.S. after March 2020. Very few cases of B/Yamagata were reported globally in 2020, and those reported after March 2020 were all live attenuated influenza vaccine (LAIV) virus [11]. This surveillance data, along with other NIRC and global surveillance data, was vital for researchers, epidemiologists, vaccine manufacturers, and policymakers, as it would contribute to changing future influenza vaccines from quadrivalent to trivalent formulations [11].

**Figure 4:**
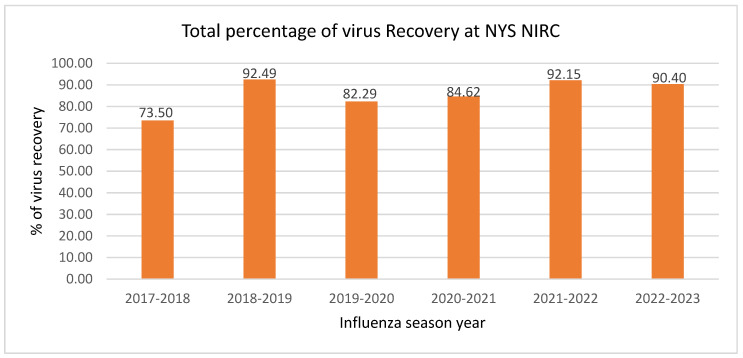
Virus recovery rates from culture of influenza clinical specimens received at the NYS NIRC VIP laboratory across the six seasons in the study.

One of the most important tasks of the VIP NIRC laboratory is to propagate influenza viruses to larger volumes to allow for phenotypic characterization. Per CDC guidance for seasons spanning 2017 through 2022, all clinical specimens submitted were inoculated into cells for culture; however, this was reduced to 50% of clinical specimens for the 2022–2023 season. Following inoculation, the T-75 cm^2^ flasks were monitored for cytopathic effect (CPE), and at the designated harvest time, hemagglutination (HA) assays were performed to determine the virus HA titer. Based on virus subtype and titer, a calculation of percentage recovery of virus isolates from clinical specimens was generated. The growth of viruses in culture depends on factors such as virus infectivity and permissiveness of cells, and the replicative capacity of the virus in cell culture [12]. Additionally, the quality of the specimen at collection, the timing of collection during a patient’s infection, and the conditions under which it is stored and transported have significant effects on the successful recovery of the virus. Proper specimen handling and storage are essential to prevent degradation, contamination, or loss of viral integrity. Figure 4 shows the overall percentage recovery of all clinical specimens received each season at the NYS NIRC VIP lab during the study period, with 2018–2019 having the highest percentage recovery (92.5%) among all six seasons, with an average recovery of 85.9%.

**Figure 5:**
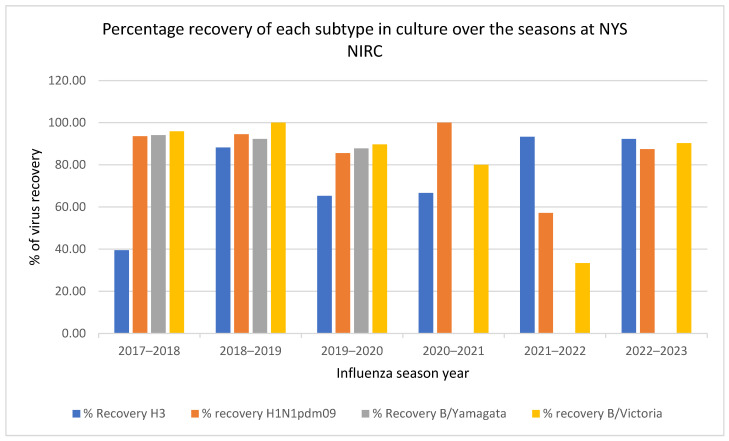
Percentage recovery of influenza viruses by type and subtype or lineage in the NYS NIRC, across the six seasons in the study.

Virus recovery varied among the studied seasons and with subtype, as shown in Figure 5. Overall, recovery rates for influenza B viruses were better than those for influenza A. Further, percentage recoveries of the B/Victoria lineage viruses were better than those for B/Yamagata viruses. Among influenza A positive clinical specimens, A/H1N1pdm09 produced better recovery than A/H3N2. Both B/Victoria and A/H1N1pdm09 had the highest (100%) percentage recovery during the 2018–2019 and 2020–2021 seasons, respectively, while B/Victoria had the lowest (33.33%) in season 2021–2022, and A/H3N2 had 39.5% recovery, the lowest in season 2017–2018. Recovery rates for each week were calculated by the total number of specimens that had at least one subsample with an isolated type of “grown,” divided by the total number of specimens inoculated. As per the standardized protocol from the CDC, an isolated type of “grown” was decided based on HA titer of the specimen. For example, in the case of Influenza B and A/H1N1pdm09, any specimen with an HA titer of 8 or more was considered grown, and for A/H3N2, any titer of 1 or more was considered grown.

**Figure 6:**
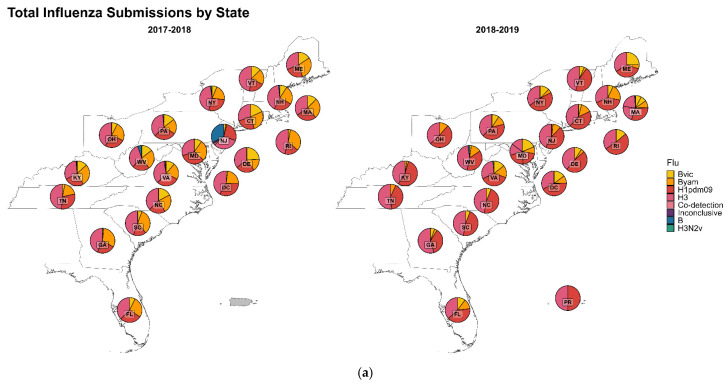

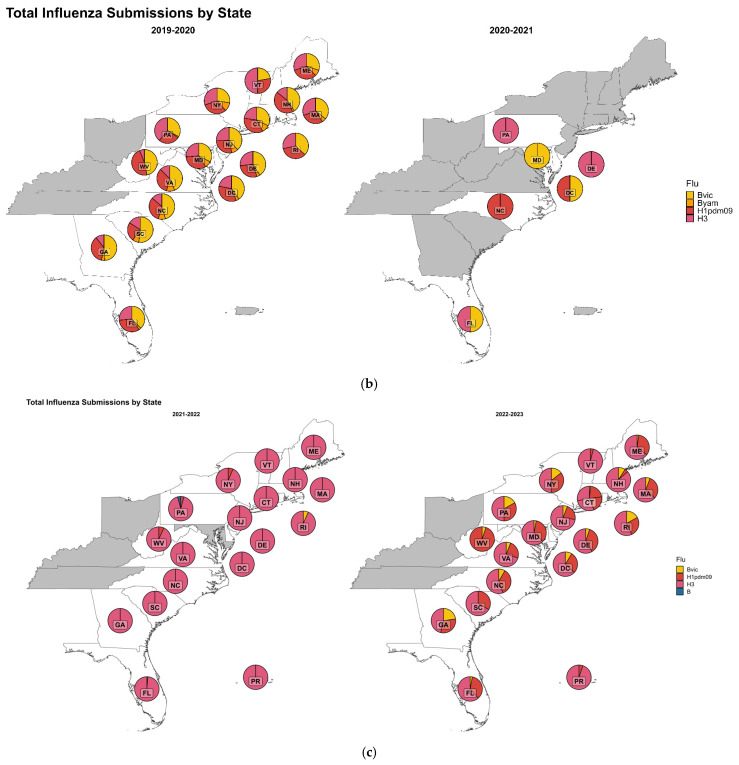
The geographically mapped data in (**a**–**c**) show the predominant circulating influenza subtypes and the variation in number from submitting states for the six seasons studied. The two-letter abbreviations in the maps represent different states in the U.S.A. ME = Maine, VT = Vermont, NH = New Hampshire, CT = Connecticut, MA = Massachusetts, RI = Rhode Island, NY = New York, NJ = New Jersey, DE = Delaware, DC = District of Columbia, MD = Maryland, PA = Pennsylvania, WV = West Virginia, VA = Virginia, NC = North Carolina, SC = South Carolina, GA = Georgia, FL = Florida, KY = Kentucky, TN = Tennessee, and PR = Puerto Rico.

The number of submitting states for NYS NIRC varied across the seasons. Prior to the COVID-19 pandemic, submissions followed a common trend, but as the COVID-19 pandemic peaked during 2020–2021, influenza activity waned to the lowest of all recent records. As shown in Figure 6, only six states submitted a total of 13 clinical specimens to the NYS NIRC that year. Also, B/Yamagata gradually disappeared from circulation for the following two seasons. As the COVID-19 pandemic progressed in subsequent years, influenza activity slowly increased and resumed usual levels during the 2022–2023 season. The number of submissions per state per season is listed in detail in Supplemental Tables S1 and S2.

## 4. Discussion

A few years after the 2009 A/H1N1 pandemic, three NIRCs were established in the U.S., one each in California, New York State, and Wisconsin, to contribute surveillance data for a more comprehensive picture of the antigenic, genetic, and antiviral susceptibility properties of influenza viruses circulating or emerging in the U.S. The establishment of the U.S. NIRCs provided substantial additional influenza surveillance data and virus isolates for the CDC’s national surveillance program, facilitating more informed decision-making for influenza guidance and policy and for candidate vaccine virus selection.

The availability of cultured viruses from clinical specimens remains an extremely important component of the surveillance program, providing material for multiple phenotypic analyses, including antigenic characterization, human serology studies, and biological measures of drug susceptibility. Additionally, the resulting virus isolates, and residual clinical specimens may also be selected as reference viruses used for additional reagent production (e.g., ferret antisera or diagnostic test controls) or as starting material for the development of future vaccine candidates. Access to clinical specimens and, moreover, virus isolates have become increasingly difficult in an era when at-home testing is becoming more common, shipping costs have increased, and virus culture laboratories in clinical settings are uncommon. In this landscape, the work of the culture laboratories in the NIRCs provides an extensive and important source of material for these purposes.

From the data generated at the NYS NIRC during the six influenza seasons reported in this study, it is evident that seasons varied in terms of the number of submissions, circulating subtypes of influenza, and percentage recovery of viruses. Overall, the predominant circulating type during all seasons remained influenza A, with A/H3N2 being more commonly dominant than any other subtypes or influenza B lineages, except for season 2019–2020 when the B/Victoria lineage dominated the season. Notably, from 2020 to 2021 onwards, B/Yamagata remained absent from any submissions and has not been reported in surveillance data within the U.S. or globally for five years. This important finding in the face of extensive influenza surveillance worldwide fueled the decision to remove it as a component of vaccines in 2024, returning both northern and southern hemisphere vaccines to a trivalent rather than quadrivalent formulation.

It was apparent that influenza activity was following normal seasonal trends before the COVID-19 pandemic, but as pandemic activity peaked and immediately after, circulating influenza reduced to an unprecedented low level. This dramatic impact was also seen on the circulation of other respiratory viruses during this time [13]. While changes in testing priorities and available resources may have influenced the earliest reports of low influenza activity, studies across the globe have shown consistent reductions in influenza activity, which is believed to be a consequence of the many actions taken to mitigate the spread of SARS-CoV-2. During this season of extremely low influenza activity, the number of clinical specimens submitted to the NYS NIRC, which usually exceeded 1000 per season, dropped to only 13 submissions.

A few limitations should be recognized when considering the conclusions drawn from this study. Specimen submissions were specifically requested as a ratio of influenza subtypes. Therefore, data is not representative of the circulating ratio, nor is the purpose of NIRCS. Recovery rates of viruses depend on many factors such as culturability and properties of viruses in the specimen, specimen storage conditions prior to shipping, environmental and culture conditions, physiological states of host cells, etc. As with all NIRC programs, comprehensive findings are dependent on submissions from states for which we are responsible. While we sincerely appreciate the shipment of these specimens, current operational demands and local outbreaks within the state laboratory’s jurisdiction restrict the laboratory’s capacity to optimally participate in national surveillance.

As we moved into the post-pandemic phase, influenza activity returned to pre-pandemic levels, with ongoing surveillance and vaccination efforts again becoming essential for continued effective management of the disease. The most recent influenza season has presented as one of the most severe in many years, with high case counts and the highest influenza-associated pediatric fatality rate since the 2009 pandemic [14]. As public health measures continue to adapt, it is essential to remain alert and prepared for changes in influenza viruses to mitigate their spread, protect vulnerable populations, and minimize the burden on health care systems.

## Data Availability

The original contributions presented in this study are included in the article/Supplementary Material. Further inquiries can be directed to the corresponding author.

## Supplementary Materials

The following supporting information can be downloaded at https://www.mdpi.com/article/10.3390/idr18040071/s1: Table S1: Influenza sample submissions to NYS NIRC, Table S2: Influenza sample submissions to NYS NIRC.
