# Supplementary material for: Influenza Virus Isolation for Public Health Surveillance Before, During, and After the COVID-19 Pandemic: Experiences from the New York State National Influenza Reference Center Laboratory
Source: Infect Dis Rep. 2026 Jul 10;18(4):71. doi: 10.3390/idr18040071 (PMC13398334; doi:10.3390/idr18040071)
Supplement: Supplementary file 1 [file idr-18-00071-s001.zip › idr-4269256-supplementary.pdf]

infectious disease reports

MDPI

Supplemental Table S1: Influenza sample submissions to NYS NIRC.

Influenza sample submission							
		Season					
State	Subtype	2017-2018 N=1,376	2018-2019 N=1,204	2019-2020 N=876	2020-2021 N=1,149	2021-2022 N=535	2022-2023 N=13
Connecticut		N=82	N=50	N=52	-	N=42	N=68
	Bvic	15 (18.3%)	2 (4.0%)	17 (32.7%)	-	-	2 (2.9%)
	Byam	22 (26.8%)	7 (14.0%)	5 (9.6%)	-	-	-
	H1pdm09	20 (24.4%)	22 (44.0%)	18 (34.6%)	-	-	28 (41.2%)
	H3	25 (30.5%)	19 (38.0%)	12 (23.1%)	-	42 (100.0%)	37 (54.4%)
	Co-detection	-	-	-	-	-	1 (1.5%)[1]
D.C.		N=48	N=61	N=50	N=6	N=56	N=57
	Bvic	1 (2.1%)	9 (14.8%)	20 (40.0%)	3 (50.0%)	-	5 (8.8%)
	Byam	11 (22.9%)	6 (9.8%)	2 (4.0%)	-	-	-
	H1pdm09	16 (33.3%)	26 (42.6%)	17 (34.0%)	3 (50.0%)	-	16 (28.1%)
	H3	20 (41.7%)	20 (32.8%)	11 (22.0%)	-	56 (100.0%)	36 (63.2%)
Delaware		N=82	N=67	N=49	N=1	N=39	N=118
	Bvic	20 (24.4%)	6 (9.0%)	20 (40.8%)	-	-	6 (5.1%)
	Byam	13 (15.9%)	3 (4.5%)	2 (4.1%)	-	-	-
	H1pdm09	20 (24.4%)	32 (47.8%)	14 (28.6%)	-	-	43 (36.4%)
	H3	29 (35.4%)	26 (38.8%)	13 (26.5%)	1 (100.0%)	39 (100.0%)	69 (58.5%)
Florida		N=149	N=146	N=106	N=2	N=78	N=104
	Bvic	11 (7.4%)	15 (10.3%)	40 (37.7%)	1 (50.0%)	-	3 (2.9%)
	Byam	40 (26.8%)	19 (13.0%)	3 (2.8%)	-	-	-
	H1pdm09	42 (28.2%)	58 (39.7%)	35 (33.0%)	-	1 (1.3%)	37 (35.6%)
	H3	56 (37.6%)	54 (37.0%)	28 (26.4%)	1 (50.0%)	77 (98.7%)	64 (61.5%)
Georgia		N=60	N=55	N=47	-	N=3	N=97
	Bvic	1 (1.7%)	3 (5.5%)	23 (48.9%)	-	-	22 (22.7%)
	Byam	19 (31.7%)	2 (3.6%)	2 (4.3%)	-	-	-
	H1pdm09	14 (23.3%)	20 (36.4%)	17 (36.2%)	-	-	30 (30.9%)
	H3	26 (43.3%)	30 (54.5%)	5 (10.6%)	-	3 (100.0%)	45 (46.4%)
Kentucky		N=67	N=44	-	-	-	-
	Bvic	9 (13.4%)	1 (2.3%)	-	-	-	-
	Byam	19 (28.4%)	1 (2.3%)	-	-	-	-
	H1pdm09	17 (25.4%)	24 (54.5%)	-	-	-	-
	H3	21 (31.3%)	18 (40.9%)	-	-	-	-
	Co-detection	1 (1.5%)[2]	-	-	-	-	-
Maine		N=64	N=56	N=40	-	N=6	N=48
	Bvic	10 (15.6%)	14 (25.0%)	12 (30.0%)	-	-	1 (2.1%)
	Byam	19 (29.7%)	3 (5.4%)	3 (7.5%)	-	-	-
	H1pdm09	15 (23.4%)	20 (35.7%)	13 (32.5%)	-	-	16 (33.3%)
	H3	20 (31.2%)	19 (33.9%)	12 (30.0%)	-	6 (100.0%)	31 (64.6%)
Maryland		N=92	N=88	N=61	N=1	-	N=67
	Bvic	9 (9.8%)	26 (29.5%)	20 (32.8%)	1 (100.0%)	-	2 (3.0%)
	Byam	25 (27.2%)	12 (13.6%)	5 (8.2%)	-	-	-
	H1pdm09	30 (32.6%)	29 (33.0%)	20 (32.8%)	-	-	23 (34.3%)
	H3	28 (30.4%)	20 (22.7%)	16 (26.2%)	-	-	42 (62.7%)
	Co-detection	-	1 (1.1%)[1]	-	-	-	-
Massachusetts		N=49	N=60	N=39	-	N=21	N=68
	B	-	1 (1.7%)	-	-	-	-
	Bvic	6 (12.2%)	6 (10.0%)	14 (35.9%)	-	-	4 (5.9%)
	Byam	13 (26.5%)	8 (13.3%)	2 (5.1%)	-	-	-
	H1pdm09	13 (26.5%)	26 (43.3%)	11 (28.2%)	-	-	21 (30.9%)
	H3	17 (34.7%)	18 (30.0%)	12 (30.8%)	-	21 (100.0%)	43 (63.2%)
	Co-detection	-	1 (1.7%)[3]	-	-	-	-
New Hampshire		N=54	N=57	N=42	-	N=31	N=22
	B	1 (1.9%)	-	-	-	-	-
	Bvic	5 (9.3%)	4 (7.0%)	17 (40.5%)	-	-	2 (9.1%)
	Byam	13 (24.1%)	12 (21.1%)	1 (2.4%)	-	-	-
	H1pdm09	13 (24.1%)	23 (40.4%)	18 (42.9%)	-	-	1 (4.5%)
	H3	22 (40.7%)	18 (31.6%)	6 (14.3%)	-	31 (100.0%)	19 (86.4%)
New Jersey		N=76	N=57	N=60	-	N=33	N=52
	B	25 (32.9%)	-	-	-	-	-
	Bvic	2 (2.6%)	1 (1.8%)	23 (38.3%)	-	-	3 (5.8%)
	Byam	1 (1.3%)	6 (10.5%)	5 (8.3%)	-	-	-
	H1pdm09	20 (26.3%)	26 (45.6%)	17 (28.3%)	-	-	11 (21.2%)
	H3	25 (32.9%)	24 (42.1%)	15 (25.0%)	-	33 (100.0%)	38 (73.1%)
	Co-detection	3 (3.9%)[2]	-	-	-	-	-

[1] H1pdm09 and H3
[2] Inconclusive
[3] H3 and Bvic

Supplemental Table S2: Influenza sample submissions to NYS NIRC

Influenza sample submission							
		Season					
State	Subtype	2017-2018<br>N=1,376	2018-2019<br>N=1,204	2019-2020<br>N=876	2020-2021<br>N=1,149	2021-2022<br>N=535	2022-2023<br>N=13
New York		N=94	N=81	N=73	-	N=31	N=76
	B	1 (1.1%)	-	-	-	-	-
	Bvic	6 (6.4%)	10 (12.3%)	20 (27.4%)	-	-	11 (14.5%)
	Byam	19 (20.2%)	4 (4.9%)	8 (11.0%)	-	-	-
	H1pdm09	26 (27.7%)	39 (48.1%)	23 (31.5%)	-	2 (6.5%)	27 (35.5%)
	H3	41 (43.6%)	28 (34.6%)	22 (30.1%)	-	29 (93.5%)	38 (50.0%)
	H3N2v	1 (1.1%)	-	-	-	-	-
North Carolina		N=70	N=34	N=44	N=2	N=16	N=36
	Bvic	12 (17.1%)	2 (5.9%)	20 (45.5%)	-	-	3 (8.3%)
	Byam	17 (24.3%)	-	4 (9.1%)	-	-	-
	H1pdm09	15 (21.4%)	16 (47.1%)	14 (31.8%)	2 (100.0%)	-	13 (36.1%)
	H3	26 (37.1%)	16 (47.1%)	6 (13.6%)	-	16 (100.0%)	20 (55.6%)
Ohio		N=53	N=35	-	-	-	-
	Bvic	4 (7.5%)	-	-	-	-	-
	Byam	13 (24.5%)	4 (11.4%)	-	-	-	-
	H1pdm09	14 (26.4%)	18 (51.4%)	-	-	-	-
	H3	22 (41.5%)	13 (37.1%)	-	-	-	-
Pennsylvania		N=62	N=75	N=64	N=1	N=48	N=118
	B	-	-	-	-	2 (4.2%)	-
	Bvic	9 (14.5%)	7 (9.3%)	21 (32.8%)	-	-	20 (16.9%)
	Byam	12 (19.4%)	9 (12.0%)	1 (1.6%)	-	-	-
	H1pdm09	18 (29.0%)	31 (41.3%)	17 (26.6%)	-	2 (4.2%)	39 (33.1%)
	H3	22 (35.5%)	28 (37.3%)	25 (39.1%)	1 (100.0%)	44 (91.7%)	59 (50.0%)
	Inconclusive	1 (1.6%)	-	-	-	-	-
Puerto Rico		-	N=10	-	-	N=29	N=19
	H1pdm09	-	5 (50.0%)	-	-	-	1 (5.3%)
	H3	-	5 (50.0%)	-	-	29 (100.0%)	18 (94.7%)
Rhode Island		N=33	N=29	N=35	-	N=45	N=88
	Bvic	1 (3.0%)	4 (13.8%)	13 (37.1%)	-	3 (6.7%)	15 (17.0%)
	Byam	11 (33.3%)	-	-	-	-	-
	H1pdm09	6 (18.2%)	15 (51.7%)	12 (34.3%)	-	-	23 (26.1%)
	H3	15 (45.5%)	10 (34.5%)	10 (28.6%)	-	42 (93.3%)	50 (56.8%)
South Carolina		N=18	N=17	N=25	-	N=4	N=30
	Bvic	1 (5.6%)	1 (5.9%)	13 (52.0%)	-	-	-
	Byam	6 (33.3%)	-	2 (8.0%)	-	-	-
	H1pdm09	3 (16.7%)	8 (47.1%)	6 (24.0%)	-	-	10 (33.3%)
	H3	8 (44.4%)	8 (47.1%)	4 (16.0%)	-	4 (100.0%)	20 (66.7%)
Tennessee		N=37	N=43	-	-	-	-
	Bvic	1 (2.7%)	-	-	-	-	-
	Byam	7 (18.9%)	3 (7.0%)	-	-	-	-
	H1pdm09	11 (29.7%)	18 (41.9%)	-	-	-	-
	H3	18 (48.6%)	22 (51.2%)	-	-	-	-
Vermont		N=40	N=31	N=18	-	N=14	N=25
	Bvic	5 (12.5%)	2 (6.5%)	4 (22.2%)	-	-	-
	Byam	8 (20.0%)	1 (3.2%)	-	-	-	-
	H1pdm09	8 (20.0%)	14 (45.2%)	5 (27.8%)	-	-	1 (4.0%)
	H3	19 (47.5%)	14 (45.2%)	9 (50.0%)	-	14 (100.0%)	24 (96.0%)
Virginia		N=71	N=63	N=39	-	N=10	N=17
	B	-	1 (1.6%)	-	-	-	-
	Bvic	8 (11.3%)	9 (14.3%)	17 (43.6%)	-	-	1 (5.9%)
	Byam	15 (21.1%)	10 (15.9%)	4 (10.3%)	-	-	-
	H1pdm09	21 (29.6%)	24 (38.1%)	13 (33.3%)	-	-	4 (23.5%)
	H3	25 (35.2%)	19 (30.2%)	5 (12.8%)	-	10 (100.0%)	12 (70.6%)
	Co-detection	2 (2.8%)[1]	-	-	-	-	-
West Virginia		N=75	N=45	N=32	-	N=29	N=39
	B	4 (5.3%)	1 (2.2%)	-	-	-	-
	Bvic	11 (14.7%)	1 (2.2%)	15 (46.9%)	-	-	2 (5.1%)
	Byam	19 (25.3%)	6 (13.3%)	-	-	-	-
	H1pdm09	17 (22.7%)	17 (37.8%)	15 (46.9%)	-	2 (6.9%)	37 (94.9%)
	H3	24 (32.0%)	20 (44.4%)	2 (6.2%)	-	27 (93.1%)	-

[1] Inconclusive
